# Medicine

**Published:** 1895-12

**Authors:** J. Michell Clarke


### Progress of the Medical Sciences.

### MEDICINE.

The recent literature of Epilepsy is considerable. Bleuler[1] found in the brains of twenty-six epileptics—fifteen males and eleven females—examined by him that there was always a distinct hypertrophy of the cortical neuroglia fibres lying between the pia mater and the outermost layer of tangential

[1] *München. med. Wchnschr.*, 1895, xlii. 769.

nerve-fibres. The glia fibres were increased in number and thickness, had a tendency to form bands running in a parallel direction, and often formed "whorls" around the entering vessels. This change was spread over the brain-mantle generally, but there were places in which it could not be observed: the author does not enter into the question as to whether any part of the brain was especially affected. The intensity of this *gliosis* was not proportional to the duration of the disease. In two of the patients a gross lesion was the cause of the epilepsy. Other histological changes were not certainly proved: glia cells were not increased in number, but often appeared pigmented and shrunken. Changes were noted in the ganglion cells, but it was difficult to say how much was due to epilepsy and how much to atrophy. The small pyramidal cells showed no extensive destructive changes. Disease in the vessels (atheroma and thickening of coats) was found in some, but was absent in most of the brains. For the purpose of control, fifty-four brains were examined from patients dying in the asylum of various forms of mental and nervous disease other than epilepsy. Gliosis was only present in fifteen of these, and the characters of the change were different from those found in epilepsy. The author admits that the above described changes may be concomitant with or consecutive to, and not causative of, epilepsy.

Todorski has investigated the condition of the circulation in the brain during the epileptic paroxysm.[1] The arterial and venous blood-pressure, and that of the cerebro-spinal fluid, were estimated during epileptiform attacks induced in dogs by faradic stimulation of the cortex cerebri or venous injection of absinthe. In non-curarised dogs the arterial blood-pressure rose some seconds after faradic irritation of the cortex was begun, and was accompanied by a rise in the venous pressure. In the stage of tonic contraction a continued rise of the arterial pressure occurs, whilst the venous quickly follows it and reaches its acme at the beginning of clonic contractions. The latter remains at the highest point some seconds, whilst the arterial pressure sinks earlier. The venous pressure returns to the normal at the end of coma. In animals curarised to avoid the hindrance to the circulation of the blood from muscular contractions, the same results were obtained even when the vago-sympathetic nerve was divided. The curve of pressure of the cerebro-spinal fluid follows that of the blood-pressure, and attains its highest point at the commencement of clonic contractions. It remains at this point for some time and then gradually falls—the rise often outlasting that of the blood-pressure. The author concludes from this that the rise of blood-pressure in the brain during the fit results in an increased outflow of lymph.

With regard to the question of the reflex origin of epilepsy, Dr. P. C. Knapp[2] thinks that in view of the anatomical changes

[1] *Centralbl. f. innere Med.*, 1895, xvi. 220.

[2] *Am. J. M. Sc.*, 1895, cx. 413.

(neuroglia-sclerosis) found in the brain-cortex, it is not possible to attribute epileptic attacks to astigmatism or errors of refraction, as has been done by some observers. There is some reason for believing that astigmatism is commoner in epileptics than in healthy persons, though in the latter refractive errors are very common; but even if this be so, astigmatism may be a mark of degeneracy rather than an etiological cause. A critical examination of cases of reflex epilepsy would certainly eliminate many of those reported under this head. "A considerable number of them are undoubtedly cases of hysteria, and in others the dependence of the epilepsy upon the alleged source of irritation is certainly doubtful. Nevertheless the thesis that a remote irritation may give rise to convulsive seizures is supported by the well-known experiments of Brown-Séquard, . . . and by a limited number of authentic cases in man. In most of these cases the source of the epilepsy has been of traumatic origin, and on removal of the irritation the epilepsy has ceased. These cases, however, are distinctly rare." In the cases of epilepsy coming under his own observation, he thinks that less than two per cent. could be assigned to such a cause. In a discussion[1] on this paper, Dr. G. E. de Schweinitz said that he doubted that true epilepsy is ever produced by errors in refraction, but that he was quite sure that convulsive seizures were modified and sometimes checked for long seasons by the use of proper glasses to correct such defects. The evident conclusion was that while we may not believe the extravagant assertions that have been promulgated with reference to the effect of eye-strain, we know that it must be subdued, for the same reason that the defective functions of any other organ must be put into proper order, to ensure successful treatment. Dr. Guy Hinsdale mentioned two cases of reflex epilepsy: one from carious teeth; the other from a diseased testicle, the result of an injury.

Writing of that form of epilepsy which makes its first appearance late in life, Dr. Maupaté says[2] that in only from 15 to 20 per cent. of cases, epilepsy appears after the age of 30, and is then due, not to hereditary predisposition, but to such agents as syphilis, alcoholism, malaria, and injuries. Clinically the symptoms are those of the more common form; but mental troubles are more frequent, and the onset of dementia may be rapid. The author notes the frequency of malaria amongst his patients. Sympson[3] published two cases of epilepsy first appearing in old age. In one patient the first attack occurred at the age of 73, and had the ordinary characters. There was no impairment of intelligence; and the patient lived to be 78. In the other case the first attack also came on at the age of 73; death took place at 74; and after each attack there was a

[1] *J. Nerv. & Ment. Dis.*, 1895, xxii. 266.

[2] *Ann. méd.-psych.*, Juillet, 1895, p. 53. Abstract in *Rev. neurol.*, 1895, iii. 558.

[3] *Brit. M. J.*, 1894, i. 1070.

permanent failure of intelligence. Cristiani[1] has made some observations on the late appearance of epilepsy in the insane. He had seen seven cases. This form of epilepsy occurs in very chronic cases of mental disease; the fits are generally isolated and separated by long intervals, the convulsions are general, and psychical changes of "epileptic character" are present. Pathologically he found a lepto-meningitis with sclerosis and atrophy of the cortex.

In a paper entitled "De la Nutrition chez les Épileptiques," Dr. Kraınski[2] gives the results of observations on eighteen epileptics during a period of 112 days. He states that (1) the body-weight is generally diminished, sometimes increased, after the fits. This diminution of weight is greatest in the *status epilepticus.* (2) The quantity of urine is increased on the day of the fit; its specific gravity is either unaltered or diminished. (3) The quantity of uric acid is increased to an appreciable extent after the fit. For one or two days, or sometimes only for some hours, before the fit it is diminished. In some cases the onset of a fit can be foretold from the diminution in the excretion of uric acid. (4) The amount of phosphoric acid is increased after a fit, but not so rapidly as that of uric acid. (5) There is no change in the excretion of chlorides. (6) Albumen and sugar are absent. The author leaves the question open as to whether the increased excretion of uric acid is the cause or the effect of the fit.

Some two years ago Prof. Flechsig advised the treatment of epilepsy by the administration of opium in large doses for a period of six weeks. Beginning with half-a-grain, the dose is gradually increased until the patient takes fifteen grains daily, in doses of three to four grains; the maximum dose should be reached by the end of the first week, and the opium is to be stopped suddenly at the end of six weeks, and bromide, in thirty-grain doses four times a day, administered for some considerable time, and the amount then slowly diminished. Dr. Joseph Collins[3] gives a year's experience of this treatment. He tried it on fifty patients drawn from hospital, private, and dispensary practice, and sums up his conclusions as follows: "(1) The plan suggested by Flechsig is not a specific in the treatment of epilepsy. (2) In almost every case in which this plan of treatment has been tried there has been a cessation of the fits for a greater or lesser time. (3) A relapse generally occurs in a period varying from a few weeks to a few months. (4) The frequency of fits after the exhibition of opium is, for the first year at least, lessened more than one half. (5) The attacks occurring after the relapse are much less severe in character than those that the patient has been accustomed to having. (6) This plan of treatment is particularly valuable in ancient and intractable cases. (7) In recent cases of idiopathic epilepsy it cannot be

[1] *Arch. di psichiat., etc.*, xvi. i, ii. Abstract in *Rev. neurol.*, 1895, iii. 564.

[2] *Rev. neurol.*, 1895, iii. 285. [3] *Med. Rec.*, 1895, xlvi. 355.

recommended. (8) The opium plan of treatment is an important adjuvant to the bromide plan as ordinarily applied. (9) The opium acts symptomatically, and merely prepares the way for and enhances the activity of the bromides and other therapeutic measures. (10) This plan of treatment permits the use of any other substances which are known to have a beneficial action in epilepsy." Axel Holmberg[1] reports a case of epilepsy in which this treatment was carried out. The patient was put under two courses of opium. During the first course symptoms of opium poisoning appeared, and the dose had to be reduced: the patient remained free from bad fits for two months, they then recurred, and a second course was undertaken; no fits then occurred for three and a half months, when the patient had three, at which time the report concludes. I have treated three cases of severe chronic epilepsy on this plan: in all, the tolerance of the patients for the large doses of opium given was remarkable. For a variable time after the treatment the fits were much less frequent and less severe. Afterwards in one patient who suffered from very frequent attacks of *petit mal* and also from severe fits about once a week, the result has been that the severe fits are less violent and only occur occasionally, whilst the attacks of *petit mal* remain unaltered in frequency. In the second patient there is some degree of improvement in the severity and frequency of the fits. The third patient, after an interval of amelioration, became as bad as ever. This treatment could not be undertaken without close supervision of the patient, and seems most suitable for chronic cases in which the bromides alone have failed. Lui[2] reports three cases treated by this method. In two patients the fits were lessened during the opium treatment; in the third they were more frequent and intense. In one case the opium had to be discontinued for a week, owing to intolerance of the drug. The fits ceased at once when the bromide treatment was begun—in two patients, to reappear after two months, but much reduced in frequency and severity—in one case they have not recurred after four months. Lui has also treated ten patients by Bechterew's method described below, with good results as to diminution in intensity and duration of fits. He prefers this method of treatment to Flechsig's.

Bechterew[3] advocates the addition of adonis vernalis to the bromides, with the object of increasing the blood-pressure and narrowing the lumen of the vessels, seeing that in an epileptic attack vaso-motor changes, resulting in an active hyperæmia of the brain, take place. He prepares an infusion of adonis vernalis of 2–3.75 grammes of the drug in 180 c.cc. water, and adds 7·5–11.5 grammes of bromide. Of this 4–6 teaspoonfuls, or even eight teaspoonfuls, are administered daily. He claims for this

[1] *Neurol. Centralbl.*, 1895, xiv. 647.
[2] *Brit. M. J.*, 1895, ii., Epitome p. 79, from *Riv. sper. di freniat.*, xxi. ii, iii.
[3] *Neurol. Centralbl.*, 1894, xiii. 838.

combination that not infrequently the fits are checked, and in other cases are decreased in number and severity.

Walling[1] recommends the use of bromide of gold, and finds that the best effects are produced by combination with arsenic. He used a solution, ten drops of which contained gr. $\frac{1}{32}$ each of the tribromide of gold and the oxybromide of arsenic. In several cases of epilepsy this solution proved to be more efficacious than any other drug.

Moeli[2] employs atropine in a dose of $\frac{1}{120}$ grain twice a day for a period of seven to eight weeks, in epileptics in whom bromide produces no effect. The number of cases of genuine epilepsy so treated was thirty-three. During the administration of atropine the bromides were continued. In about one-third the beneficial effects of the combined bromide and atropine could be seen in diminished frequency and severity of the fits. In two men and three women, in whom previous treatment by large doses of bromide had been unsuccessful, the attacks were stopped for several weeks. This is in accord with the common experience that in certain cases of epilepsy the addition of belladonna to the bromides gives good results.

C. Féré[3] remarks on the difficulty of deciding whether an epileptic is cured unless he is placed under the closest supervision. So that it is often a matter of doubt whether to continue the administration of the bromides after the fits have ceased, especially as many patients have remained well after a year's use of the remedy. But this is a result which can neither be anticipated nor promised; and when the bromide is effectual, it should be taken continuously. The bromides act more by suspending than by curing the disease, which cannot be considered to be abolished even after a year has passed free from fits. The continued administration of bromide is of undoubted value and, if it is properly administered, is free from injurious consequences. Intolerance can only be ascertained by its effects in individual cases. Tolerance of the drug is in a very large number of patients considerable.

With regard to the more recently introduced bromides of strontium and zinc, the former seems to possess little advantage over the bromides of the alkalies, except that it is of service in cases where there is much irritability of the digestive organs, or in which the depressing effects of bromide of potassium are marked. Dr. J. Collins says[4] that there is so much uncertainty about the bromide of zinc, both in regard to its dose and action, that he leaves it out of consideration.

The same author observes with regard to antifebrin and antipyrin—the use of the latter in epilepsy, in conjunction with

1 *Med. & Surg. Reporter*, 1895, lxxii. 121. Abstract in *J. Nerv. & Ment. Dis.*, 1895, xxii. 257.
2 *Therap. Monatsh.*, 1894, viii. 436. Abstract in *J. Nerv. & Ment. Dis.*, *ibid.*
3 *Rev. de méd.*, 1895. Abstract in *Centralbl. f. innere Med.*, 1895, xvi. 741.
4 *Loc. cit.*

ammonium bromide, was introduced by Wood—that they have been more extensively employed in epilepsy than most new drugs, and in many cases with gratifying results. "The general consensus of opinion in reference to antipyrin and antifebrin is that the former is preferable, and when given in from five to ten-grain doses three to four times daily it materially enhances the value of the bromides in the treatment of idiopathic epilepsy, particularly in the less aggravated clinical forms."

Of borax—which is undoubtedly efficacious both as an aid to treatment by bromides and as a substitute for bromides in cases in which the latter have no effect, being perhaps especially valuable in controlling night attacks—Féré says[1] that it does not possess, as has been contended by some observers, therapeutic properties superior to those of the bromides. It may also give rise to accidents, not only of mild but of severe and even fatal character, and these may occur from the administration of small doses. Intestinal and cutaneous antisepsis tends to prevent them. He enumerates the following troubles from the abuse of borax: *A*. Mild—nausea, vomiting, diarrhœa; dryness of skin and of mucous membranes (loss of epithelium, fissures on lips or tongue, &c.); loss of hair and of the nails; various skin eruptions, followed by desquamation, and sometimes accompanied by cachexia, swelling, and œdema; a peculiar form of cachexia, accompanied by cutaneous troubles. Painful swelling of the sterno-mastoids, which passed off in from two to three weeks, was observed in two cases. If the drug is discontinued, all these troubles cease. *B*. Grave—nephritis, with consequent albuminuria, œdema, and uræmia. Two of his patients had uræmia: one of them died, and *post-mortem* parenchymatous nephritis was found.

Dr. Collins[2] considers the value of nitro-glycerine in epilepsy slight. In a few cases where fits of mild character or attacks of *petit mal* occurred with great frequency, I have found nitroglycerine, given in addition to the bromides, of decided value. It should be administered three or four times a day, and the dose gradually increased up to 5 minims of 1 per cent. solution.

Dr. Cortot[3] recommends the use of oxide of zinc in doses of 1 gramme to 2¼ grammes (? in twenty-four hours) for adults. He has employed it with good effect in six cases.

Dr. E. D. Bondurant[4] draws the following conclusions from observations on one hundred epileptics with mental disorder during a period of three years: (1) Borax, antipyrin, acetanilide, phenacetin, and many other so-called anti-epileptic remedies, are, with the exception of rare cases, without influence on the course of epilepsy with mental disorders. (2) $\beta$-naphthol is occasionally of service, but not more so than purgatives. (3) The bromides may postpone the attacks, but do more harm than

[1] *Semaine méd.*, 1894, xiv. 497. Abstract in *Rev. neurol.*, 1895, iii. 48. [2] *Loc. cit.*

[3] Thèse de Paris, 1894. Abstract in *Rev. neurol.*, 1895, iii. 47.

[4] *Am. J. Insan.*, 1894-5, li. 23.

good. (4) During maniacal attacks isolation may be necessary. Sedatives should very seldom or never be employed. (5) In the *status epilepticus* bleeding is the best remedy; chloral the most reliable drug.

A. Eulenburg[1] reports a case in which the cortical arm-centre was excised in a man of 28 years, who had suffered from fits since the age of 11. There were symptoms of paralysis in the face and right arm, and the fits began in the arm. After the operation, the fits remained absent for seven months; but then mild attacks began to return. Eulenburg observes that the so-called traumatic and partial epilepsies are not easy to identify, and do not offer certain prospect of cure by operation. On the other hand, cases of idiopathic epilepsy are not to be altogether excluded from surgical treatment. The failure of operative procedures rests less on insufficient diagnosis and technique than on the limits set by the nature of the disease.

The following case, quoted by E. Leyden, shows the necessity, in the investigation of what appear to be undoubted cases of idiopathic epilepsy, of enquiry as to previous injury, and of carefully examining the skull for evidence of such past injuries, which may have occurred many years before and have been forgotten. A man of 30 years of age[2] who had had a fracture of the right side of the skull when 4 years old, was suddenly seized in 1892 with left-sided convulsions: these had recurred since. In 1894 severe pain came on in the right side of the head, with numbness and weakness in the left arm and leg, followed by paralysis of these limbs. He became drowsy, and, as an area of depression was discovered in the bone over the right central convolutions, he was trephined over this spot. Nothing was found but some thickening of the connective tissues between the bone and the dura mater. The symptoms were quickly relieved after the operation, slight dragging of the left leg being the only symptom left three months afterwards. It is especially noteworthy in this case that the epileptiform attacks first came on twenty-four years after the accident, and that with such slight anatomical changes the clinical symptoms were so severe and so promptly relieved by operation.

J. MICHELL CLARKE.

### SURGERY.

The surgical treatment of Graves's disease has been much discussed lately, and a consensus of opinion obtains that much can be done by surgical means for the relief of this affection. One of the most scientific articles on this subject was that by T. Wette,[3] who defines Graves's disease as a reflex neurosis

[1] *Berl. klin. Wchnschr.*, 1895. Abstract in *Centralbl. f. innere Med.*, 1895, xvi. 670.
[2] *Berl. klin. Wchnschr.*, 1894, xxxi. 839. Abstract in *Centralbl. f. innere Med.*, 1895, xvi. 48.
[3] *Arch. f. klin. Chir.*, 1892, xliv. 652, 764.

